# Physiological and clinical variables identify ARDS classes and therapeutic heterogeneity to glucocorticoids: a retrospective study

**DOI:** 10.1186/s12890-023-02384-w

**Published:** 2023-03-21

**Authors:** Qingbo Liao, Yufan Pu, Xiaoer Jin, Zhiwei Zhuang, Xiaowen Xu, Xiaoqiang Ren, Gaoqing Liu, Qi Ding

**Affiliations:** 1 The Affiliated Suzhou Hospital of Nanjing Medical University, 26 Daoqian Road, Suzhou, 215000 PR China; 2 Gusu School, Nanjing Medical University, 458 Shizi Road, Suzhou, 215000 PR China; 3 Department of Emergency, Suzhou Municipal Hospital, 26 Daoqian Road, Suzhou, 215000 PR China; 4 First Affiliated Hospital of Suzhou University, 899 Pinghai Road, Suzhou, 215000 PR China

**Keywords:** ARDS, Glucocorticoids, Therapeutic heterogeneity, Subphenotypes, Latent profile analysis

## Abstract

**Objective:**

We aimed to identify new classes in acute respiratory distress syndrome (ARDS) using physiological and clinical variables and to explore heterogeneity in the effects of glucocorticoid therapy between classes.

**Methods:**

Using the Medical Information Mart for Intensive Care-IV database, we identified patients with ARDS. Potential profile analysis was used to identify classes with physiological and clinical data as delineating variables. Baseline characteristics and clinical outcomes were compared between classes. The effect of glucocorticoid treatment was explored by stratifying by class and glucocorticoid treatment.

**Results:**

From 2008 to 2019, 1104 patients with ARDS were enrolled in the study. The 2-class potential analysis model had the best fit (P < 0.0001), with 78% of patients falling into class 1 and 22% into class 2. Additional classes did not improve the model fit. Patients in class 2 had higher anion gap, lactate, creatinine, and glucose levels and lower residual base, blood pressure, and bicarbonate compared with class 1. In-hospital mortality and 28-day mortality were significantly higher among patients in class 2 than those in class 1 (P < 0.001). Heterogeneity of glucocorticoid treatment was observed, stratified by class and treatment, with no significant effect in class 1 (P = 0.496), increased mortality in class 2 (P = 0.001), and a significant interaction (P = 0.0381). In class 2, 28-day survival was significantly lower with glucocorticoid treatment compared with no hormone treatment (P = 0.001).

**Conclusion:**

We used clinical and physiological variables to identify two classes of non-COVID-19-associated ARDS with different baseline characteristics and clinical outcomes. The response to glucocorticoid therapy varied among different classes of patients.

**Supplementary Information:**

The online version contains supplementary material available at 10.1186/s12890-023-02384-w.

## Introduction

Acute respiratory distress syndrome (ARDS) is a complex and heterogeneous clinical syndrome defined on the basis of clinical criteria. In the 50 years since Ashbaugh and colleagues first described ARDS, substantial progress has been made in identifying causative factors of the syndrome and improving ventilatory support for patients [1]. Although the mechanisms that cause ARDS are now better understood than ever before and decades of progress have been made in the experimental understanding of ARDS and in preclinical studies to identify potentially effective new therapies, most clinical trials of ARDS have not shown a benefit in terms of mortality [2, 3]. In recent years, an explanation for the failure of these clinical trials has been proposed, namely, that ARDS has widely defined criteria, which inevitably leads to clinical and biological heterogeneity [4]. The large amount of clinical and biological heterogeneity in this syndrome is widely recognized as an important barrier to identifying effective treatments for ARDS.

To reduce heterogeneity, recent studies have focused on identifying specific ARDS subphenotypes or endophenotypes of patients who either have a higher risk of disease-related outcomes (prognostic enrichment) or respond differently to treatment (predictive enrichment), allowing for accurate drug trials in critical care [5, 6]. In 2014, Calfee and colleagues conducted a review of two reanalyses of baseline clinical and plasma biomarker data from patients with ARDS in randomized controlled trials (RCTs; the ARMA study and the ALVEOLI study) and identified two ARDS subgroups: a hyperinflammatory phenotype characterized by higher levels of inflammatory biomarkers, shock, metabolic acidosis, and mortality, and a second, non-hyperinflammatory phenotype [7]. In addition, Famous et al. used latent class analysis to reanalyze baseline clinical and biomarker data from 1000 patients with ARDS in the RCT “Fluid and Catheterization Trial” (FACTT) and identified two different phenotypes that were similar but responded differently to fluid management strategies [8]. A number of subsequent ARDS subphenotype studies have also identified two distinct ARDS phenotypes with similar clinical outcomes (mortality, ventilator-free days, organ failure-free days and days in the intensive care unit [ICU]) [9–11]. Importantly, with respect to the idea that the temporal evolution of ARDS may influence subphenotype assignment, studies have shown that both ARDS phenotypes remain stable over 3 days [12]. Predictive enrichment has also been identified for both phenotypes in terms of differential treatment response to positive end-expiratory pressure strategies [7], fluid therapy [8], and simvastatin [10, 11].

These subphenotypes were derived in independent unsupervised examinations of clinical trial populations, including LCA of clinical and biomarker variables [13], and observational cohort studies with cluster analysis of biomarker data only. Researchers have shown that ARDS subphenotypes can be accurately identified with parsimonious classifier models using three (interleukin [IL]-8, bicarbonate, and protein C) or four variables (IL-8, bicarbonate, protein C, and vasopressin use), thereby facilitating the identification of ARDS phenotypes for use in clinical trials and practice [14].

In previous studies, the identification of ARDS phenotypes has been limited to patients enrolled in RCTs, so it is unclear whether these phenotypes can be generalized to the broader ARDS population. The identification of these phenotypes has been dependent on the quantification of study biomarkers, with plasma biomarkers being a key component of phenotype identification. To translate phenotypic findings into patient care, we need to be able to analyze and measure biomarkers in real time or in the field to classify patients into subphenotypes. There are currently no commercially available immediate or real-time quantifiable analyses of biomarkers. In this context, we wanted to test whether the use of clinical data (physiological and clinical variables) available at the bedside as class definition variables in the LPA model would lead to the identification of new potential classes.

Based on the results of randomized clinical trials, there are no specific drugs that have been shown to be effective in the treatment of ARDS. According to the pathological features of ARDS, glucocorticoids have generated considerable interest for their ability to reduce lung and systemic damage in patients with ARDS due to their potent anti-inflammatory and anti-fibrotic properties [15]. For example, it has been shown that long-term methylprednisolone administration in patients with refractory ARDS is associated with improved lung injury and multiple organ dysfunction scores and reduced mortality [16]. In another RCT, although methylprednisolone improved cardiopulmonary physiology, the results did not support its routine use in the treatment of persistent ARDS. Moreover, starting methylprednisolone therapy more than two weeks after the onset of ARDS may increase the risk of death. [17]. In addition, early administration of dexamethasone may reduce the duration of mechanical ventilation and overall mortality in patients with established moderate-to-severe ARDS [18]. In a recent meta-analysis of RCTs summarizing the use of corticosteroids in all examined patients with ARDS, it was shown that the use of corticosteroids may reduce mortality in these patients. This effect was consistent between patients with COVID-19 and non-COVID-19-related ARDS, corticosteroid type, and dose [19]. Different corticosteroid regimens have been tested in patients with ARDS but without conclusive results. Notably, given the lack of use of simvastatin drugs for different subtypes of ARDS in the HARP-2 study [10] and more recently in a COVID-19-related ARDS subgroup, subphenotypes had different therapeutic responses to corticosteroids [20]. We observed that trials testing corticosteroids did not use subphenotypes of non-COVID-19-associated ARDS. Thus, it remains to be determined whether glucocorticoids have different therapeutic responses and provide different survival benefits for different ARDS subphenotypes identified in the non-COVID-19 population.

We hypothesized that all patients with ARDS in the Medical Information Mart for Intensive Care (MIMIC)-IV database could be identified in two different patient classes with different clinical outcomes using their easily accessible bedside clinical data (physiological and clinical variables) as class-defining variables in the LPA model. Furthermore, in the two classes of ARDS identified, glucocorticoids may have different treatment responses, and their benefit may be more evident in one class.

## Methods and materials

### Data sources

This was a retrospective study in which data were obtained from a large intensive care database of multi-parameter intelligent monitoring, MIMIC-IV version 1.0 (https://physionet.org/content/mimiciv/1.0/). The MIMIC-IV database contains clinical information of patients in the Beth Israel Deaconess Medical Center (BIDMC) ICU between 2008 and 2019. The database has been approved by the Massachusetts Institute of Technology and BIDMC Institutional Review Board [21]. We were granted access to the database for data extraction following successful completion of the National Institutes of Health web-based training course and the Protecting Human Research Participants Examination (Fellowship No. 9,650,688, Record ID 39,539,950). Informed consent was not required as all health data were anonymous.

### Study population

The total number of inpatients listed in the MIMIC-IV database from 2008 to 2019 was 523,740, and we selected patients with a diagnosis of ARDS for analysis by combining the diagnostic information recorded in the database with the Berlin criteria. Patient diagnoses were determined according to the International Classification of Diseases criteria (ICD-9-CM, ICD-10-CM) [22]. The following exclusion criteria were used: (1) patients with multiple repeat hospitalizations (only data from the first hospitalization were included); (2) patients aged < 18 years at the time of ICU admission; (3) ICU length of stay < 48 h; and (4) patients with massive data loss.

### Data collection

We used the Navicat Premium 15 platform and Structured Query Language to extract identifiers (ID, ICU admission number), demographics (age, sex, height, weight), vital signs (temperature, heart rate, respiratory rate, blood pressure, SPO2, urine output), arterial blood gas parameters (lactate, PO2 PCO2, alveolar-arterial oxygen partial pressure difference, PaO2/FiO2 ratio, residual base, total CO2, anion gap, bicarbonate), invasive ventilation parameters (tidal volume, plateau pressure, PEEP, minute ventilation, oxygen concentration), laboratory parameters (white blood cells, hemoglobin, platelets, hematocrit, glucose, potassium, sodium, chloride, calcium, blood creatinine, blood urea nitrogen), and ARDS. The data on risk factors (such as pneumonia, sepsis, trauma, and malabsorption) were analyzed using vital signs, ventilator parameters, and laboratory parameters generated within 24 h of ICU admission. For some variables with multiple measurements within 24 h, maximum and minimum values were evaluated according to clinical characteristics (Additional file 1). Laboratory parameters such as albumin, CRP, and bilirubin were not included in the analysis due to excessive missing data. Medication use (including vasoactive drugs and glucocorticoids) was also extracted for the entire hospitalization, where vasoactive drugs included use 24 and 48 h before admission to the ICU and for the entire hospitalization, and glucocorticoids were used for the entire ICU admission, and the route of administration was intravenous.

The following outcome indicators were identified in this study: in-hospital mortality, days on ventilation, ventilator-free days, ICU length of stay, length of stay, and 28-day mortality.

### Latent profile analysis (LPA)

LPA is similar to latent class analysis (LCA) in that it assumes that the data contain many unobserved groups or classes and determines group heterogeneity by mixture modelling to determine the best-fit model for a set of data [23]. When used to deal with continuous variables, it is called LPA. Potential class-based methods have been widely used in other disciplines, medicine, and related ARDS studies [7, 8, 10, 20].

Data on continuous physiological and clinical variables in demographics, vital signs, invasive respiratory parameters, blood gas analysis parameters, and laboratory parameters were assessed as class-defining variables for LPA, excluding all categorical variables. Partially similar demographics, vital signs, and laboratory parameters were used as defining variables, according to previous studies [7, 8, 10, 20]. Unlike previous studies, we also included variables not used in previous LCA studies, such as residual base, total CO2, and anion gap. All variables are easily available at the bedside. Among variables, urine volume was subjected to unit changes to reduce variance exceeding the upper limit of analytical detection, and highly correlated variables were excluded. The full set of class-defined variables used for LPA (31 continuous variables) is shown in Additional file 1. Clinical outcome variables and categorical variables were not included in the model.

To determine the optimal number of classes, we sequentially fitted class models from 1 to 5 using LPA and then performed these five model evaluations to determine the best class model. The model fit evaluation metrics were entropy, information metrics, and significance tests. The entropy value (Entropy) evaluates the classification accuracy and takes a value in the range of 0–1, with values closer to 1 indicating more accurate classification (0.8 indicates a classification accuracy of more than 90%). Information indicators included Akaike information criterion (AIC), Bayesian information criterion (BIC), and adjusted Bayesian information criterion (aBIC); the smaller the statistical value, the better the model fit. Significance tests including the likelihood ratio test (bootstrap-based; BLRT) and LMR were used to compare the difference in fit between k − 1 and k classes; a significant p-value indicated that the k-class model outperformed the k-1 class model. To estimate the model parameters, all continuous variables were zero-mean normalized, with all means normalized to 0 and standard deviations of 1. Once the optimal number of classes was determined using the metrics, each individual was assigned to the class to which they belonged according to the maximum posterior categorical probability. Correlations between classes and clinical outcomes were then tested using a method developed by Lanza [24] and stratified by class and glucocorticoid treatment modality, to determine if there was a differential treatment effect based on potential classes. The LPA process was performed using Mplus8.

### Statistical analysis

For baseline characteristics, continuous variables are expressed as mean and standard deviation or median and interquartile range (IQR), depending on whether they were normally distributed; categorical variables are expressed as number and percentage. The *t*-test, chi-square test (χ^2^), Kruskal–Wallis test, and Wilcoxon rank-sum test were used for comparisons of baseline characteristics between classes, according to the nature of the variables.

The chi-square test for mortality (i.e., proportion of deaths) and the Wilcoxon rank-sum test for correlation between class assignment of ventilator-free days and clinical outcomes were used. To test for interactions between treatment and class assignment, we used logistic regression of mortality and performed post hoc subgroup analyses.

To assess the prognostic value of individual variables and to explore treatment interactions between different classes and glucocorticoid exposure, a logistic regression model was used to estimate the dominance ratio, with in-hospital mortality as the dependent variable. For 28-day mortality, Kaplan–Meier survival curves were plotted according to different strata.

Missing data were < 20% for all variables, and for variables with 5% < proportion missing < 20%, missing data were subjected to multiple interpolations using the chained equation (MICE) method [25]. The outlier values were replaced with the 99th and 1st percentiles. The percentage of missing data is described in detail in Additional file 1. Additional file 2 shows the distribution of each parameter before and after multiple interpolations of the missing data.

Data were collapsed and combined and statistically analyzed using Stata SE16 (StataCorp LLC, College Station, TX, USA) and IBM SPSS version 26.0 (IBM Corp., Armonk, NY, USA). P values < 0.05 were considered statistically significant.

## Results

**Fig. 1:**
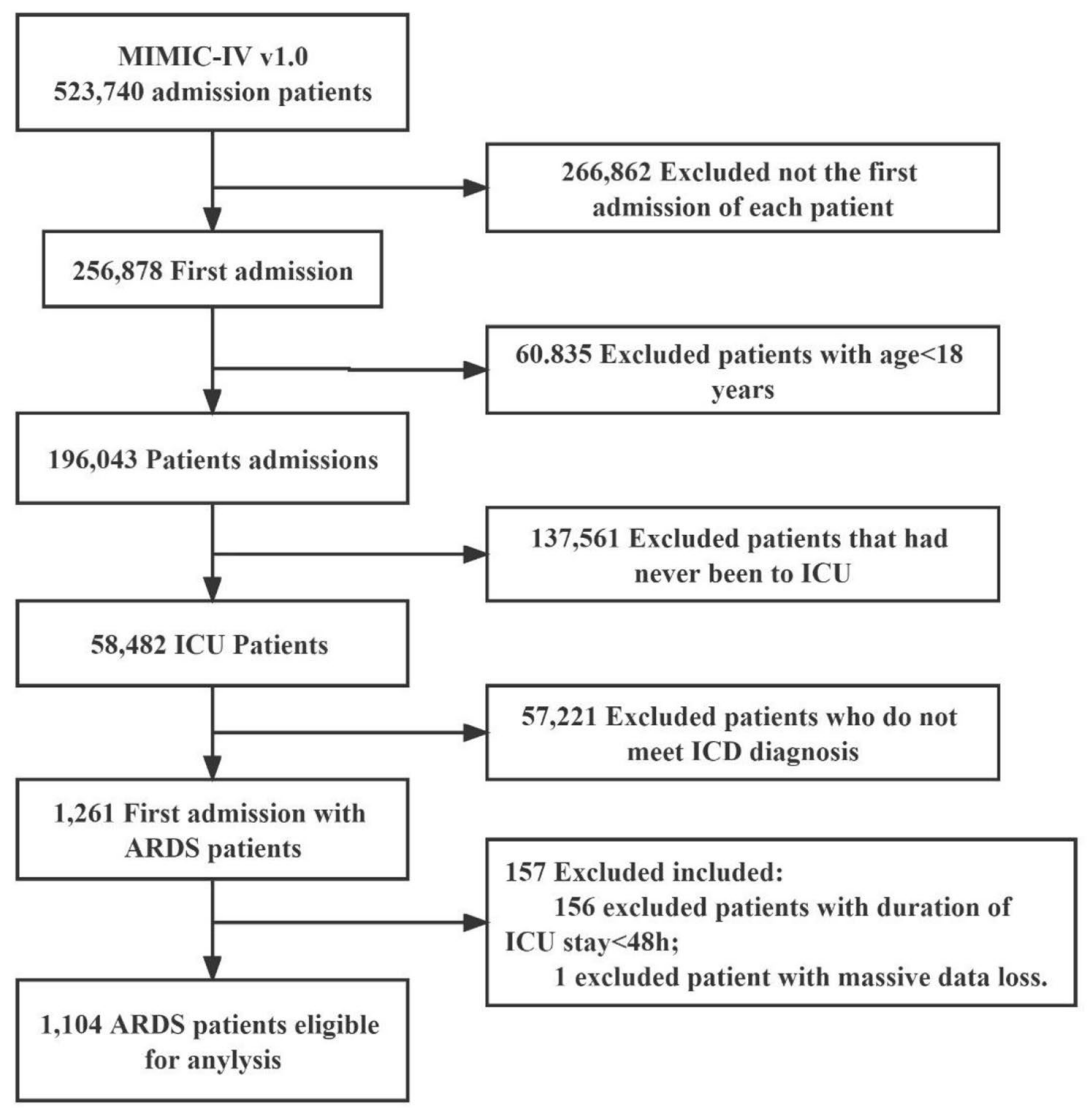
*Study flowchart. MIMIC, Medical Information Mart for Intensive Care*

During the study period, 523,740 hospitalized patients were included in the MIMIC-IV database, and 1261 patients admitted to the ICU for the first time with ARDS were included based on diagnostic criteria. Of these, 157 patients were excluded from the study. Of these, 156 were excluded due to ICU stays of less than 48 h and 1 patient was excluded due to missing key respiratory parameters, resulting in the inclusion of 1104 patients in the cohort (see flow chart, Fig. 1).

### Clinical characteristics

Of the 1104 patients (Table 1), the mean age was 63 (± 18) years, 603 patients (54.6%) were women, and most patients (63.0%) were non-Hispanic White. Regarding risk factors for ARDS, 319 patients with ARDS had pneumonia, 378 experienced trauma (including surgery), 247 had sepsis, 14 experienced aspiration, and 382 had other risk factors (including acute hypoxic respiratory failure, and pancreatitis). The median tidal volume of invasive breathing during patients’ stay in the ICU was 410 mL (345.5–482.5 mL), which may be related to the protective ventilation strategy adopted. Vasoactive drugs were required in 217 (19.7%) patients throughout the entire hospital stay and were administered to 119 (10.8%) patients within 24 h of ICU admission and 150 (13.6%) patients within 48 h. Glucocorticoids were administered to 172 (15.6%) patients. The median length of stay in the ICU was 9 days (IQR, 5–15), the median length of stay in the hospital was 15 days (IQR, 9–23), and the median days of ventilation were 3 (IQR, 1–6).

**Table 1:** Baseline characteristics of included patients and clinical characteristics and biological differences between the 2 classes

Baseline Characteristic	All patients (n = 1104)	Class1 (n = 866)	Class2 (n = 238)	P value
Age, year	63 ± 18	64 ± 18	61 ± 18	0.0258
Gender, n (%)				0.14
female	501(45.4%)	403(46.54%)	98(41.18%)	
male	603(54.6%)	463(53.46%)	140(58.82%)	
Height, cm	168.8 ± 10	168.7 ± 10	169.0 ± 10	0.6295
Weight, kg	85.8 ± 24	85.4 ± 24	87.3 ± 24	0.2809
The risk of ARDS, n (%)				< 0.001
pneumonia	319(28.9%)	240(27.71%)	79(33.19%)	
trauma	378(34.2%)	203(23.44%)	52(21.85%)	
sepsis	247(22.4%)	87(10.05%)	49(20.59%)	
aspiration	14(1.3%)	9(1.04%)	3(1.26%)	
other	382(34.6%)	327(37.76%)	55(23.11%)	
Vasopressor Use, n (%)				
vaso_use_24h^#1^	119(10.8%)	44(5.08%)	75(31.51%)	< 0.001
vaso_use_48h^#2^	150(13.6%)	64(7.39%)	86(36.13%)	< 0.001
vaso_use_all^#3^	217(19.7%)	116(13.39%)	101(42.44%)	< 0.001
Corticosteroids, n (%)	172(15.6%)	125(14.13%)	47(19.75%)	0.045
Race, n (%)				0.009
White	696(63.04%)	568(65.59%)	128(53.78%)	
BLACK/AFRICAN	75(6.79%)	53(6.12%)	22(9.24%)	
HISPANIC/LATINO	45(4.08%)	31(3.58%)	14(5.88%)	
Asian	29(2.63)	24(2.77%)	5(2.10%)	
other	259(23.46%)	190(21.94%)	69(28.99%)	
PaO_2_fiO_2_ratio	161.8(98.9-246.8)	175(115–263)	113(75–193)	< 0.0001
PcO_2_, mmHg	49(43–56)	49(43–55)	52(44–60)	0.0039
PO_2_, mmHg	80(66–108)	81(68–113)	74(61–90)	< 0.0001
Aado_2_, mmHg	356.2(216.0-528.6)	324(205–492)	503(293–577)	< 0.0001
Totalco_2_, mEq/L	21(18–24)	22(20–25)	16(14–18)	< 0.0001
min volume, L/min	11.5 ± 3	11.1 ± 3	12.8 ± 3	< 0.0001
Tidal volume, mL	410(345.5-482.5)	410(341–480)	419(351–490)	0.2167
Plateau pressure, cmH_2_O	22(18–26)	21(18–25)	26(21–30)	< 0.0001
Peep, cm H_2_O	8(5–11)	7(5–10)	10(6–15)	< 0.0001
FiO_2_, %	100(60–100)	100(60–100)	100(100–100)	< 0.0001
Lactate, mg/dl	3(1.7–4.9)	2.6(1.6–3.9)	6.4(4.1–8.7)	< 0.0001
Be, mmol/L	-5(-8–1)	-3(-6-0)	-12(-15–9)	< 0.0001
Hematocrit, %	35.7 ± 6	35.4 ± 6	36.8 ± 7	0.0018
Hemoglobin, mg/dL	9.7 ± 2	9.9 ± 2	9.1 ± 2	< 0.0001
Platelets, 10^9^/L	155.5(103–216)	169(114–226)	117(73–172)	< 0.0001
Wbc, 10^9^/L	14.9(11.2–20.0)	14.1(10.9–18.8)	17.6(12.7–23.3)	< 0.0001
Glucose, mg/dL	179(146–223)	171(142–207)	233(181–301)	< 0.0001
AG, mmol/L	16(13–19)	15(13–17)	21(18–24)	< 0.0001
Bicarbonate, mmol/L	21 ± 4	22 ± 4	16 ± 4	< 0.0001
Bun, mmol/L	21(15–30)	19(14–26)	31(20–46)	< 0.0001
Calcium, mg/dL	7.9 ± 0.9	8.0 ± 0.8	7.5 ± 1.0	< 0.0001
Chloride, mmol/L	108(105–112)	108(105–111)	110(105–113)	< 0.0001
Creatinine, mg/dL	1.1(0.8–1.6)	1.0(0.8–1.3)	1.8(1.3–2.7)	< 0.0001
Sodium, mmol/L	141 ± 4	140 ± 4	142 ± 5	0.0005
Potassium, mmol/L	3.9 ± 0.5	3.9 ± 0.5	3.9 ± 0.6	0.4217
h, beats per min	108(93–123)	105(91–119)	120(105–135)	< 0.0001
Temperature, °C	37.7 ± 0.8	37.7 ± 0.8	37.7 ± 0.8	0.6690
Sbp, mmHg	85 ± 14	87 ± 14	80 ± 14	< 0.0001
RR, breaths per min	27(24–32)	27(23–31)	29(25–34)	< 0.0001
Urine output, L	1.5(0.9–2.3)	1.6(1-2.4)	1.0(0.5–1.8)	< 0.0001
Scoring systems				
SAPSII	41(33–51)	39(31–48)	49(40–62)	< 0.0001
SOFA	8(5–11)	7(5–9)	12(10–14)	< 0.0001

*Abbreviations defined: Aado*_*2*_ = *alveolar-arterial oxygen difference; PEEP* = *positive end-expiratory pressure; Be* = *residual base; AG* = *aniongap; Bun* = *urea nitrogen; HR* = *heart rate; RR* = *respiratory rate; Sbp* = *systolic blood pressure. Normally distributed data are expressed as mean* ± *standard deviation; non-normally distributed data are expressed as median (interquartile range); categorical data are expressed as n (%). p-values represent t-test for normally distributed data, Wilcoxon’s rank test for non-normally distributed data, and chi-square test for categorical variables. vaso_use_24h*_*#1*_*: patients who received vasopressor medications within the first 24 hours of admission to the ICU. vaso_use_48h*_*#2*_*: patients who received vasopressor medications within the first 48 hours of admission to the ICU. vaso_use_all*_*#3*_*: patients who received vasopressor medications at any time during ICU.*

### Potential profile analysis LPA

The 2-class potential class model provided the best model fit according to the potential profile analysis. We fitted potential class models from 2 to 5 classes. The summary information is presented in Table 2. All models had entropy values > 0.8, indicating > 90% accuracy in classification, and 2-class entropy values > 0.9, indicating > 95% accuracy in classification. Among the information metrics, the BIC decreases as the number of classes fitted increases, indicating that the model fits better as the number of classes increases. Similarly, the decrease in the AIC and aBIC also indicates that the model fits better as the number of classes increases. The 2-class model had the largest decrease in information metrics and outperformed the 1-class model whereas the significance test showed that LMR < 0.0001 and BLRT < 0.0001 for the 2-class model was significant. The 3-, 4-, and 5-class models had lower information metrics than the 2-class model, but the LMR of class 3 = 0.0730 and the class 5 LMR = 0.4354, both with P-value > 0.05. The LMR of the class 4 model = 0.0459, but the sample size of one class was too small, so the 2-class model was the best fit. In the 2-class model, 866 (78.4%) patients were assigned to class 1 and 238 (21.6%) were assigned to class 2. The mean class probability for the most likely class was 0.981 for class 1 and 0.957 for class 2 (see Additional file 3).

**Table 2:** LPA latent class model fitting information

Model	k	AIC	BIC	aBIC	Entropy	LMR	BLRT	Class probability
1	62	97216.491	97526.906	97329.979				
2	94	94612.867	95083.496	94784.929	0.912	< 0.0001	< 0.0001	0.784/0.216 866/238
3	126	93270.001	93900.845	93500.638	0.864	0.0730	< 0.0001	0.312/0.538/0.150 344/594/166
4	158	92236.822	93027.880	92526.033	0.893	0.0459	< 0.0001	0.516/0.297/0.129/0.058 570/328/142/64
5	190	91581.529	92532.801	91929.314	0.886	0.4354	< 0.0001	0.265/0.437/0.112/0.124/0.061 293/482/124/137/68

*Abbreviations: K = degrees of freedom; AIC = Akaike information criterion; BIC = Bayesian information criterion; aBIC = adjusted Bayesian information criterion, the smaller the statistical value, the better the model fit. BLRT = likelihood ratio test (Bootstrap-based BLRT); LMR = Vuong-Lo-Mendell-Rubin test.*

### Baseline characteristics of each class

**Fig. 2:**
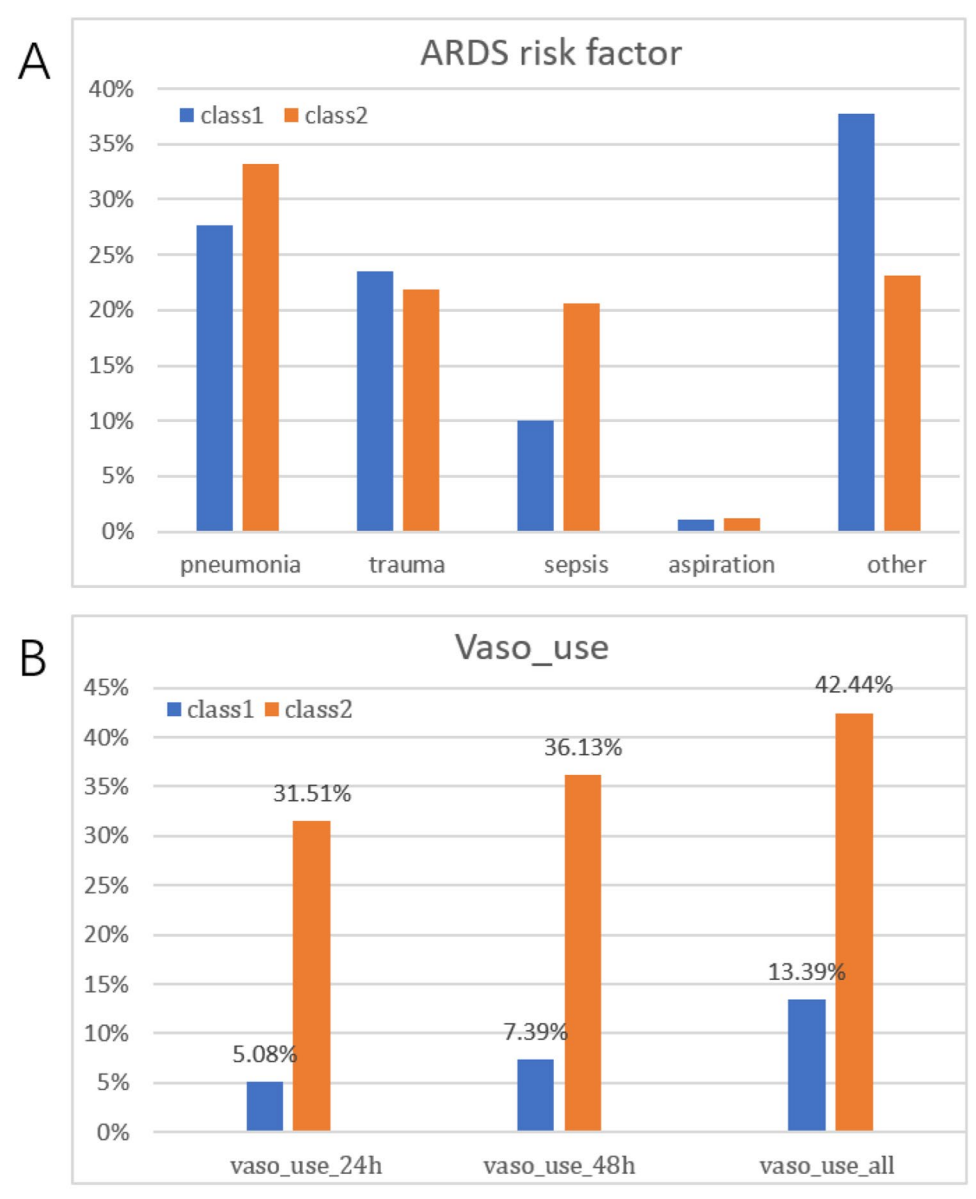
**Two classes of vasoactive drug use(A) and ARDS-related risk factors at 24 h, 48 h, and throughout hospitalization in the ICU(B).** Abbreviations: K = degrees of freedom; AIC = Akaike information criterion; BIC = Bayesian information criterion; aBIC = corrected Bayesian information criterion, the smaller the statistical value, the better the model fit. bLRT = likelihood ratio test (Bootstrap-based BLRT); LMR = Vuong-Lo-Mendell-Rubin test

**Fig. 3:**
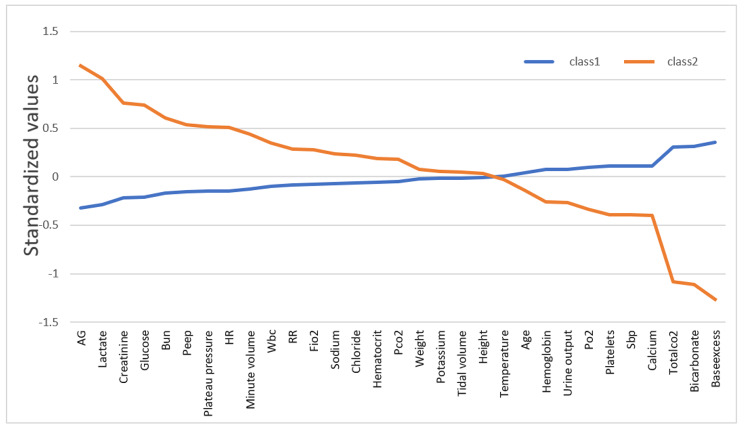
**Standardized values of continuous variables.** The continuous classes used in the latent class model define the normalized values of the variables. For the highest values in class 2, variables are sorted from left to right in descending order. Standardized values were calculated by zero-meaning the mean of the variables to 0 and specifying the standard deviation as 1. AG, anion gap; Bun, urea nitrogen; PEEP, positive end-expiratory pressure; HR, heart rate; WBC, white blood cell count; RR, respiratory rate; and SBP, systolic blood pressure

Patients were assigned to their two most likely classes based on posterior classification probabilities. For simplicity, we refer to these two classes as class 1 and class 2. There were 866 patients in class 1 and 238 patients in class 2 (Table 1). In the 2-class model, risk factors for ARDS (pneumonia, trauma, sepsis, and other, mainly acute hypoxic respiratory failure) were more common. In class 2, the proportion of pneumonia was higher. In class 1, the main risk factor was acute hypoxic respiratory failure, among others. The use of vasoactive drugs increased with hospitalization duration, from 24 to 48 h of ICU admission to the entire hospitalization period. The number of patients using vasoactive drugs was higher in class 2 compared with class 1 patients, with the proportion being > 30%, possibly indicating that class 2 patients were at greater risk of shock (Fig. 2). Among the 2-class of ARDS patients, the proportion of male patients was > 50% (53.46%; 58.82%), and the proportion of white patients in the race was > 50%. We standardized all continuous variables to a zero mean and arranged them in order of magnitude of the variable values in class 2 (Fig. 3). (Results normalized for original continuous variables were presented in the Additional file 4). Among all continuous class-defined variables, anion gap, lactate value, creatinine, residual base, bicarbonate, and total carbon dioxide differed more in class 2. In class 2, anion gap, lactate value, and creatinine were significantly higher than in class 1, whereas residual base, bicarbonate, and total carbon dioxide were significantly lower than in class 1, possibly indicating that patients in class 2 had more severe acidosis and poorer renal function than patients in class 1.

### Clinical outcomes in two potential classes

**Fig. 4:**
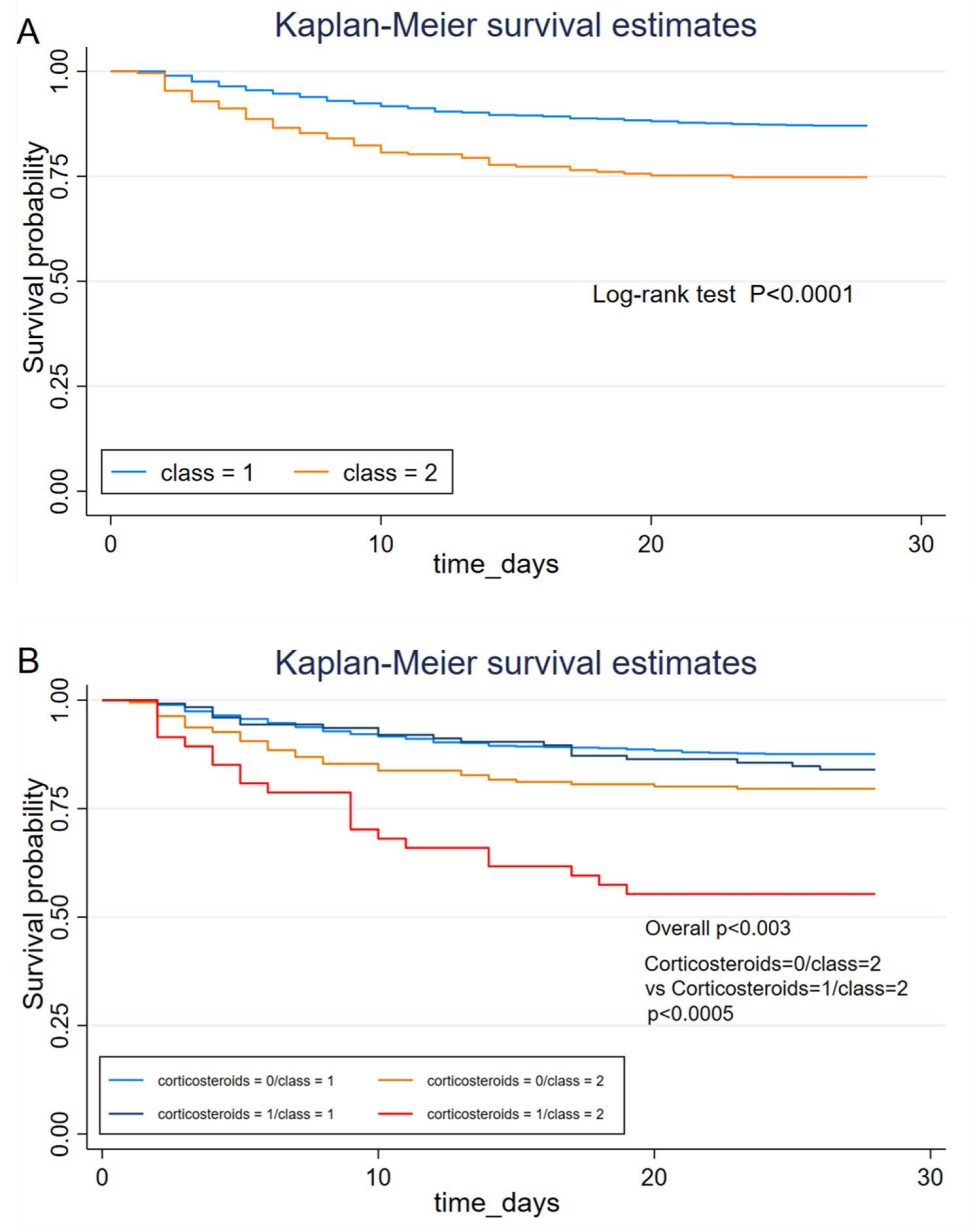
**K-M survival curves, stratified by ARDS class and glucocorticoid treatment.** Kaplan-Meier survival curves for patients with ARDS at 28 days, with class 2 patients having significantly lower survival than class 1 in panel A (P < 0.0001). panel B, stratified by ARDS class and treatment (glucocorticoid use versus no use). Curves were compared using the log-rank test. Overall p < 0.003; p < 0.0005 for class 2 patients treated with glucocorticoids versus not treated with glucocorticoids; p = 0.301 for hormone exposure in class 1 versus not

 The clinical and biological characteristics of patients in class 2 were similar to the hyperinflammatory subphenotype in a previous study [7, 8, 10]. In that study, patients in class 2 had significantly higher in-hospital mortality and 28-day mortality compared with those in class 1 (27.3% vs. 13.9%, P < 0.001; 25.3% vs. 12.9%, P < 0.001). Patients in class 2 had significantly more days of ICU stay and overall length of stay than patients in class 1 (P < 0.0001). For ventilator-free days, patients in class 2 had longer days than patients in class 1 (P < 0.0001) (see Table 3). In logistic regression, by adjusting for covariates such as age, gender, corticosteroids, vasoactive drug use, SAPS II score and SOFA score, the mortality rate in class 2 was equivalent to approximately 1.31 times that of class 1 patients (odds ratio [OR] = 1.31; P = 0.038) (Table 4). Across the cohort, although associated with an increased risk of mortality, glucocorticoid use was statistically non-significant (P = 0.094). In addition, in both class Kaplan-Meier survival curves (Fig. 4A), class 2 patients had significantly lower 28-day survival than those in class 1 (P < 0.0001)

**Table 3:** clinical outcomes of two classes

Clinical Outcome	Class1 (n = 866)	Class2 (n = 238)	P-value
Mortality (Los), n (%)	120(13.9%)	65(27.3%)	< 0.001
Mortality (28-day), n (%)	112(12.9%)	60(25.2%)	< 0.001
ICU LOS, median (25–75%)	9(5–15)	13(9–19)	< 0.001
Hospital LOS, median (25–75%)	15(9–23)	19(11–28)	< 0.001
Ventilator-Free Days, median (25–75%)	26(23–27)	24(20–26)	< 0.001

*Ventilator-free days observation until 28 days*

**Table 4:** The relationship between clinical variables and in-hospital mortality in different classes

Variables	OR	95% Conf. Interval	P value
Class2(reference 1)	1.31	(1.03–1.69)	0.038
Age	1.03	(1.02–1.05)	< 0.001
Gender(male)	0.67	(0.48–0.94)	0.022
SAPS II score	0.99	(0.97–1.01)	0.527
SOFA score	1.18	(1.11–1.25)	< 0.001
Corticosteroids	1.43	(0.94–2.19)	0.094
Vaso_use_24h^#1^	0.38	(0.06–2.34)	0.299
CC^#2^	2.11	(0.84–5.26)	0.043
CV^#3^	1.73	(0.60–4.99)	0.308

*Multivariate logistic regression. Vaso_use_24h*^#1^: *patients who received vasopressor medications within the first 24 h of admission to the ICU. CC*^#2^: *an interaction term between the classes and the vasopressor(vaso_use_24h). CV*^#3^: *an interaction term between the classes and the corticosteroids*

### Treatment response of glucocorticoids between classes

In logistic regression stratified by ARDS class and glucocorticoids, class 1 OR = 1.20 indicated that the risk of death may be increased approximately 1.2-fold with glucocorticoids in class 1 (P = 0.496), with a non-significant P-value. Class 2 OR = 3.27 indicated that the risk of death with glucocorticoid use in class 2 may be increased approximately 3.27 times (P = 0.001). The final interaction test (P = 0.0381) indicated that for in-hospital mortality, the effect of glucocorticoids was significantly different between the two classes. Glucocorticoid exposure was associated with a trend toward increased mortality in ARDS class 2 and was not significantly associated with changes in mortality in class 1 (Table 5). We observed significant differences in survival curves among patients stratified by class and treatment (Fig. 4B; overall P < 0.003). Specifically, in patients in class 2, 28-day survival was significantly lower with glucocorticoid treatment than in patients without it (P < 0.0005). In class 1, no difference was observed for glucocorticoid treatment (P = 0.301).

**Table 5:** In-hospital mortality, stratified by glucocorticoid therapy and ARDS class

	ARDS Class Inhospital expire				
	Corticosteroids_use(Yes)	Corticosteroids_use (No)	OR (95%CI)	P-Value	P for interaction
Class					0.0381
class1(n = 866)	22 /125(18%)	98 /741(13%)	1.20(0.71–2.05)	0.496	
class2(n = 238)	22/47(49%)	43/191(23%)	3.27(1.57–6.78)	0.001	

As a sensitivity analysis to determine whether general severity of illness scores could supplant class identification, we tested for interactions between SAPS II score and corticosteroids. In contrast to the analyses using class, there were no significant interactions between SAPS II score and corticosteroids for the outcomes of mortality (p = 0.809 for interactions).

## Discussion

This study aimed to verify whether using only clinical data (physiological and clinical variables) available at the bedside of patients with ARDS (excluding biomarkers) as a class definition variable could identify new potential classes. We identified two new potential classes of ARDS with different biological and clinical characteristics and clinical outcomes using clinical data as class definition variables for LPA. Patients with ARDS in class 2 had more severe acidosis, more vasoactive drug use, and worse clinical outcomes compared with those in class 1. There was a significant interaction between the two class pairs in our study and glucocorticoid therapy, with glucocorticoid use increasing mortality in class 2 in this study but not in class 1.

In this study, we performed LPA potential profiling in a larger sample size (n = 1104) of patients with ARDS than in previous studies [7, 8, 10, 20, 26]. In our study cohort, the informational metrics and VLMR and BLRT test P-values for the two classes strongly suggested that the 2-class model was the best fit in this study. Although the P-value of VLMR for the four classes = 0.0459, the 3-class model fit was not significant due to the two classes, which, together with the smaller size of the fourth class, led us to focus on the 2-class model. A possible reason for this is that the final decision on the optimal number of classes determined by the LPA model requires consideration of many different factors. Assuming that there are “X” potential classes in the data, the potential class model seeks to find the best model fit. If there are only two classes, but we fit a 3-class model, the third class will be forced in by selecting a few cases with more extreme or unique sets of values. A relatively small class provides very little information and may represent an anomaly rather than a useful finding.

We observed that the LPA class-defining variables with the largest contributions to potential classes included anion gap, base residual, lactate, bicarbonate, total carbon dioxide, and creatinine, with the contribution of bicarbonate having been reported in previous studies [7, 8, 10, 20]. These contributing clinical variables are extra-pulmonary variables that provide a greater correlation between circulatory and organ function in patients, and it can be briefly hypothesized that class 2 patients likely have poorer circulatory and organ function than class 1 patients. The possible explanation for the smaller contribution of lung-related indicators, such as respiratory parameters, to the identification of potential classes is that patients with ARDS who meet the diagnostic criteria of ARDS syndrome have less heterogeneity in the inverse of respiratory parameters.

With subsequent subgroup analysis of the two classes, we found heterogeneity in the effect of glucocorticoid therapy in both ARDS classes. Importantly for the subgroup analysis, first, we are a retrospective study, and glucocorticoid treatment was nonrandomized and could not be predetermined for planned subgroup analysis. We performed an unplanned exploratory subgroup analysis and a “robustness check”; the interaction of glucocorticoid treatment was significant across classes. Given the limitations of the study design, interpretation of the presented findings should be made with caution and limited to hypothesis generation. Second, although we noted that the proportion of glucocorticoid therapy was close in class 1 and class 2 (14% vs. 19%), we did not perform further subgroup analyses because the proportion of patients receiving each glucocorticoid was too small.

 In the present study, the effect of glucocorticoid therapy reflected heterogeneity across different subclasses of ARDS. Of the three largest clinical trials testing the benefit of glucocorticoids in critically ill patients with COVID-19, the CoDEX randomized clinical trial (COVID-19 dexamethasone), the randomized clinical trial of hydrocortisone in critically ill patients with COVID-19, and the REMAP-CAP randomized clinical trial of COVID-19 corticosteroids, no significant reduction in absolute mortality was observed [27–29]. Notably, glucocorticoid treatment in all these studies had a signal of mortality benefit. It can be speculated that treating different subphenotypes may have different therapeutic effects, which requires prospective clinical trials. In addition, regarding dexamethasone in patients hospitalized with COVID-19, a preliminary report of a reduction in 28-day mortality with dexamethasone in patients randomized to invasive mechanical ventilation or oxygen alone, but not in patients not receiving respiratory support, suggests heterogeneity in terms of disease severity [30]. In patients with COVID-19-associated ARDS, glucocorticoid exposure was associated with reduced mortality in the high inflammatory phenotype and with increased mortality in the low inflammatory subphenotype [20]. Compared with previous studies, the effect of glucocorticoid treatment in our study differed across phenotypes. Our study population of patients with non-COVID-19-related ARDS had a broader range of risk factors for ARDS and greater heterogeneity. The potential classes identified by reducing heterogeneity may differ from the subphenotypes of COVID-19-related ARDS, so their glucocorticoid treatment effects differ across classes. Although no significant effect of glucocorticoid treatment was observed among patients with relatively mild illness in our study class 1, the sample size of this population was large, the signal-to-noise ratio was small, and glucocorticoid treatment likely had a harmful effect on this small group of people. This needs to be further evaluated in the future.

Our study found that patients using glucocorticoids had a lower probability of survival. The decision to use glucocorticoid may be influenced by factors such as comorbidity, disease severity, and prior treatment, which may also affect survival. To reduce indication bias, we excluded patients who may have used corticosteroids for comorbidities and also performed a sensitivity analysis, which found no significant interaction between disease severity SAPS II score and corticosteroids on the outcome of mortality. However, we acknowledge that there may be residual confounding that cannot be fully controlled in our study, which could be a potential limitation.

The present study has several strengths. First, most of the previous studies exploring ARDS heterogeneity have been based on a combination of clinical traits and biomarkers to distinguish their heterogeneity. Biomarkers are difficult in clinical practice because they are not available promptly or are difficult to obtain. We used clinical and physiological variables easily accessible at the patient’s bedside to perform LPA and obtained two different classes of ARDS. One study identified two ARDS subtypes using clinical variables [31], but we studied inconsistent methods so that the presence of ARDS subphenotypes could be verified from different perspectives. Second, we relied on the data-driven and unbiased nature of LPA for latent class identification without considering patient clinical outcome data. We included a large sample size, in comparison with previous studies, including ARDS patients with different risk factors. In addition, there has been controversy regarding the use of glucocorticoids in patients with ARDS in terms of whether glucocorticoids have different treatment effects in different classes. In this study, we further analyzed the two different classes of ARDS obtained. In patients with ARDS in class 1, glucocorticoid use was not statistically different for in-hospital mortality. In class 2 patients, glucocorticoid use increased patient mortality, which yields hypotheses for future studies.

This study also has some limitations. Although two classes were identified, we cannot guarantee that the addition of plasma biomarker variables (e.g., sRAGE, SP-D, ANG1/2, vWF, VEGF) and pathology-related variables will yield a different phenotype [5, 32–35] as the pathology of ARDS is also variable at autopsy. Hormone therapy was measured throughout the patient’s hospitalization, and we were unable to test for heterogeneous changes in the effect of hormone therapy in a given period. In addition, we were unable to obtain follow-up data on patients over time, and their long-term mortality is unknown. Finally, the low percentage of ARDS selected in the MIMIV-IV database is also one of the limitations of our study.

 In conclusion, we identified two classes of non-COVID-19-associated ARDS using clinical and physiological variables with specifically different clinical and biological characteristics and clinical outcomes, with class 2 having a poorer clinical outcome. Differential treatment effects of glucocorticoids were observed in the different classes. Prospective clinical studies may be needed to verify the therapeutic heterogeneity of glucocorticoids in the different classes.

## Electronic supplementary material

Below is the link to the electronic supplementary material.

Supplementary Material 1

## Data Availability

The datasets generated and analyzed during the current study are not publicly available due to the need for licenses from MIT and Beth Israel Deaconess Medical Center (BIDMC) but are available from the author Qingbo Liao on reasonable request.

## List of abbreviations

ARDS: Acute respiratory distress syndrome
RCT: Randomized controlled trial
ICU: Intensive care unit
LCA: Latent class analysis
IL: Interleukin
LPA: Latent profile analysis
MIMIC: Mart for Intensive Care
BIDMC: Beth Israel Deaconess Medical Center
PaO_2_: Pressure of oxygen
FiO_2_: Fraction of inspired oxygen
PEEP: Positive end-expiratory pressure
CRP: C-reactive protein
AIC: Akaike information criterion
BIC: Bayesian information criterion
K: Degrees of freedom
aBIC: Adjusted Bayesian information criterion
BLRT: Likelihood ratio test (Bootstrap-based BLRT)
LMR: Vuong-Lo-Mendell-Rubin test
IQR: Interquartile range
Aado_2_: Alveolar-arterial oxygen difference
Be: Residual base
AG: Aniongap
Bun: Urea nitrogen
PEEP: Positive end-expiratory pressure
HR: Heart rate
WBC: White blood cell count
RR: Respiratory rate
SBP: Systolic blood pressure
OR: Odds ratio
