# Supplementary material for: Physiological and clinical variables identify ARDS classes and therapeutic heterogeneity to glucocorticoids: a retrospective study
Source: BMC Pulm Med. 2023 Mar 21;23:92. doi: 10.1186/s12890-023-02384-w (PMC10028772; doi:10.1186/s12890-023-02384-w)

**Physiological and Clinical Variables Identify ARDS Classes and Therapeutic Heterogeneity to Glucocorticoids:** **A retrospective study**

**SUPPLEMENTAL MATERIAL**

**Additional file 1.** Amount of missing data for each variable included in the analysis

**Additional file 2.** Distributions of predictors before and after multiple imputations

**Additional file 3.** The average class probability for the most likely class

**Additional file 4.** Results normalized for original continuous variables

**Additional file 1. Amount of missing data for each variable included in the analysis**

| **Variable** | **Missing data N(%)** | **Min** | **Max** |
| --- | --- | --- | --- |
| **class-defined variables** |  |  |  |
| Age | 0(0%) | 19.0 | 92.5 |
| Height | 156（14.1%） | 122 | 196 |
| Weight | 8（0.7%） | 42.5 | 220.7 |
| Heart_rate_max | 4（0.4%） | 59 | 196 |
| Temperature_max | 115（10.4%） | 31.9 | 40.4 |
| Sbp_min | 12（1%） | 18 | 150 |
| Resp_rate_max | 4（0.4%） | 13 | 65 |
| Urine output | 12（1.1%） | 0 | 9775 |
| Lactate_max | 126（11.4%） | 0.6 | 32 |
| Po2_min | 48（4.3%） | 26 | 415 |
| Pco2_max | 48（4.3%） | 25 | 173 |
| Baseexcess_min | 48（4.3%） | -36 | 19 |
| Totalco2_min | 48（4.3%） | 4 | 47 |
| Hematocrit_max | 0 | 21.1 | 60.4 |
| Hemoglobin_min | 0 | 1.7 | 16.4 |
| Platelets_min | 0 | 9 | 1039 |
| Wbc_max | 0 | 0.1 | 137.3 |
| Glucose_all_max | 1（0.1%） | 80 | 999999 |
| Aniongap_max | 1（0.1%） | 7 | 45 |
| Bicarbonate_min | 0 | 2 | 36 |
| Bun_max | 0 | 3 | 123 |
| Calcium_min | 94（8.5%） | 2.3 | 12.9 |
| Chloride_max | 0 | 87 | 135 |
| Creatinine_max | 0 | 0.2 | 15.5 |
| Sodium_max | 0 | 120 | 164 |
| Potassium_min | 1（0.1%） | 1.5 | 6.6 |
| Tidal_volume_observed_min | 102（9.2%） | 0 | 753 |
| Plateau_pressure_max | 184（16.7%） | 10 | 50 |
| Peep_max | 98（8.9%） | 0.5 | 36 |
| Fio2_max | 56（5.1%） | 30 | 100 |
| Minute_volume_max | 100（9.0%） | 2.62 | 550 |
| **other variables** |  |  |  |
| Gender | 0 | - | - |
| Ethnicity | 0 | - | - |
| Pao2fio2ratio_min | 93（8.4%） | 26 | 1160 |
| Aado2_calc_max | 93（8.4%） | -237.6 | 645 |

**Additional file 2. Distributions of predictors before and after multiple imputations**


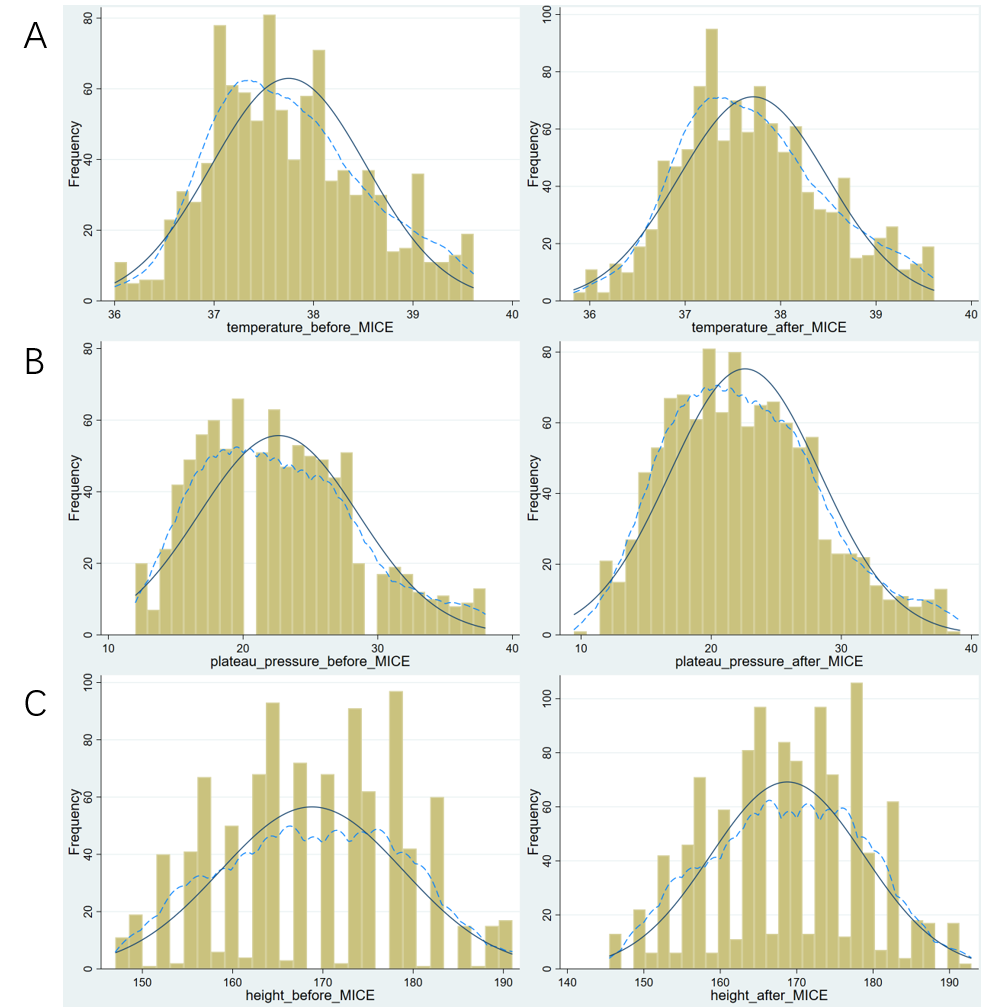


Legend: The distributions for each variable that were included in the imputation models.

**Additional file 3. The average class probability for the most likely class**

|  | 1 | 2 |
| --- | --- | --- |
| 1 | 0.981 | 0.019 |
| 2 | 0.043 | 0.957 |

Average Latent Class Probabilities for Most Likely Latent Class Membership(ROW) By Latent Class(Column).

**Additional file 4. Results normalized for original continuous variables**


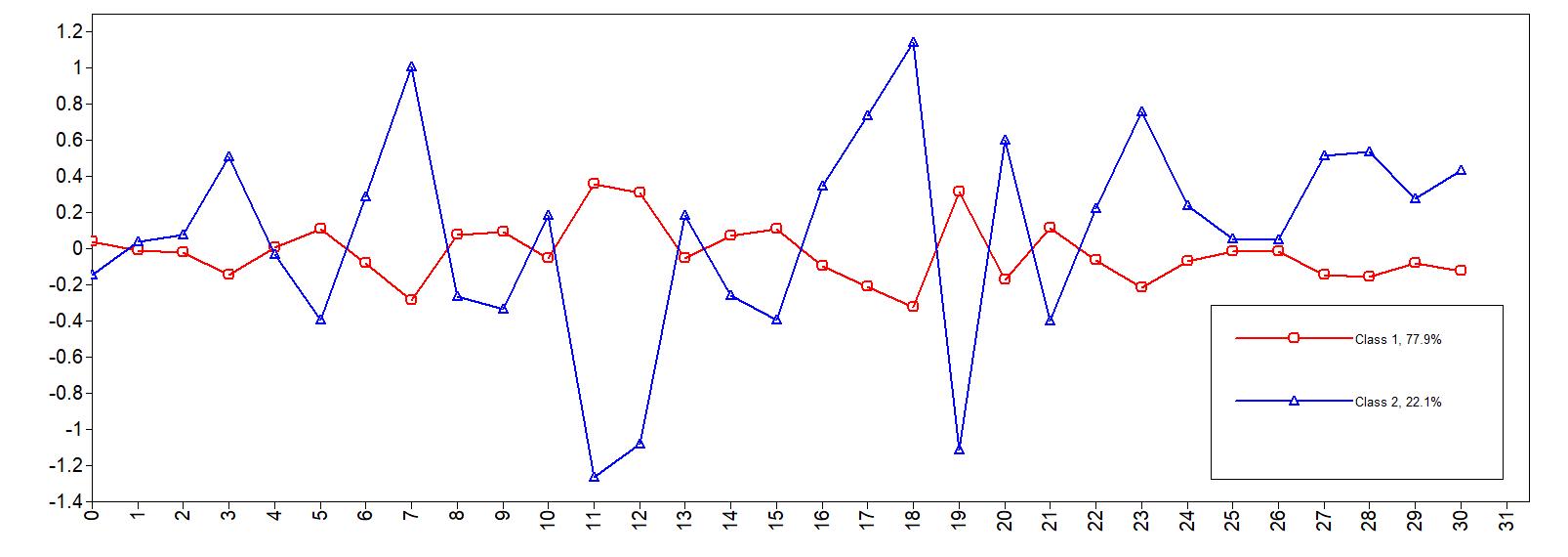

Supplement: Supplementary file 1 — Supplementary Material 1 [file 12890_2023_2384_MOESM1_ESM.docx]
